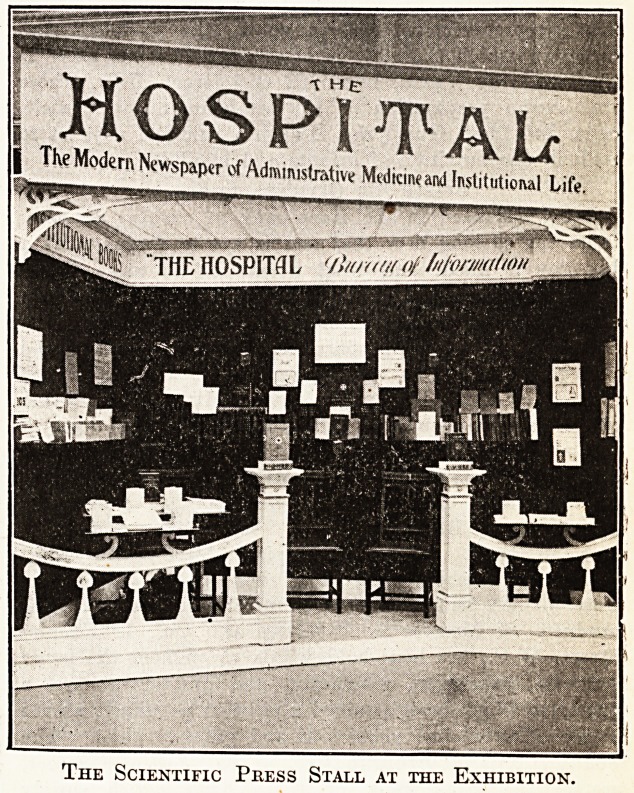# The Exhibition of the Seventeenth International Congress of Medicine

**Published:** 1913-08-16

**Authors:** 


### THE EXHIBITION OF THE SEVENTEENTH INTERNATIONAL CONGRESS OF MEDICINE.

### An Attractive Display at the Imperial Institute.

The managers of the *British and Colonial Druggist* have such a well-known and long-established reputation as organisers of the annual Medical Exhibition, usually held in Vincent Square, that it was a very happy thought on somebody's part to entrust them with the arrangements for a similar Exhibition in connection with the seventeenth International Congress of Medicine. The *British and Colonial Druggist* have acted on behalf of the Finance Committee of the Congress; and the triumphant success of this feature must be as gratifying to the Committee as it is creditable to those who have been thus entrusted with its inception and execution.

### Site and Arrangement.

Housed on the first floor of the Imperial Institute, in a fine hall and flanking transepts, the Exhibition is provided with ample space and is at the same time in the very hub and centre of the activities of the Congress. It has been visited during the time it was open, August 5 to 12, by thousands of visitors, British and foreign; and presents so many and such attractive features that we are certain they must all have received a highly favourable impression. Without exception, practically, the stalls and exhibits are of such high quality, such up-to-dateness, such indispensability, and are displayed with so much ingenuity and skill, that not only is the most favourable impression instantly created, but also it becomes a task of great difficulty to select especially notable sections for appreciation. In mentioning briefly, therefore, as many as possible of the best exhibits, it must be understood that they happened during a brief survey to catch the eye, and that no disparagement is intended of the remaining products and stalls which space prevents us from detailing.

As one mounts the fine flight of steps towards the Exhibition, one enters first a vestibule which contains the five stands of the ever enterprising firm of Burroughs Wellcome and Co., of Snow Hill, E.C. Very wisely they have not attempted to cram this vestibule with specimens of their manufactures; a large clear space is left in the centre, from which a much better view of the attractive show-cases in the corners of the hall is obtained than if too great a profusion of material had been put on show. Next we noticed the specialities of the Saccharin Corporation, 10 Arthur Street, E.C., whose pergenol and novocain are perhaps of chief importance from a pharmacological standpoint. The former of these is a solid compound of hydrogen peroxide, which dissolves immediately in water to make a neutral solution of hydrogen peroxide and boric acid. As it will keep indefinitely in dry storage, this compound is an exceedingly useful one. Novocain is, of course, the well-known local anæsthetic, such a strong favourite for both local and spinal anæsthesia.

The firm of M. Schaerer, of Berne and 41 Berners Street, W., has been before now the subject of favourable comment in these columns, especially in connection with operating tables and similar hospital necessaries. Their exhibit, just to the right as one enters the main hall, fully sustains their reputation as providers of extraordinarily ingenious, but withal practical, surgical instruments. An older friend of the hospital surgeon and theatre sister is Allen and Hanburys, many of whose exhibits bear the names of the prominent English surgeons who have devised them. Many kinds of labour-saving and efficiency promoting tools will be found at their stall, together with theatre accessories, which show how well they understand and cater for the varied needs of the modern surgical institution. Another smart stall is that of the Charles H. Phillips Chemical Co., 14 Henrietta Street, Covent Garden, W.C., so well known in connection with milk of magnesia, digestible cocoa, and similar products.

### Some Outstanding Exhibits.

Next in order are some exceedingly interesting sections. The bedsteads and sick-room furniture of J. Nesbit-Evans and Co., Floodgate Street, Birmingham, are probably household words with most of our readers, as they are in use in so many institutions and have achieved such high repute. Any to whom they are not familiar will be well advised to visit Stands 10 and 11 without fail, where every possible courtesy and attention will, we feel certain, be bestowed upon them. Conveniently next inspected should be the display of John Ward, Ltd., 246 Tottenham Court Road, W., the celebrated makers of bath-chairs, wheel-chairs, adjustable couches, and inventions of every sort for the ease and comfort of the human body. Mechanical ingenuity is seen in these chairs, combined with that luxury and durability which is so especially a feature of the best British-made goods; and it may not be amiss to explain that this firm has been established in London for nearly two hundred years. An equally fascinating exhibit, though in a different branch, is that

The Scientific Press Stall at the Exhibition.

of Messrs. Reynolds and Branson, the well-known Leeds chemists and surgical instrument makers. Here may be seen not only specimens of their latest pharmaceutical preparations, so noted for elegance and reliability, but also sphygmomanometers, tuberculin outfits, apparatus for producing artificial pneumothorax, and all sorts of similar articles, turned out with all the finish that the medical profession has come to expect from this firm. Two other admirable preparations are the familiar izal, shown by Newton, Chambers and Co., of Thornecliffe, near Sheffield, at Stall 56, and the table-waters of the Apollinaris Company, Ltd., shown at the adjoining stand. Both of these products are so well known and so much appreciated by all institutional managers that we need hardly do more than just mention their names to secure for them the attention of visitors. In the same category comes Glaxo (46 and 47 King's Road, St. Pancras), the dried milk, which has achieved such marked popularity, and has so well deserved it. Here, too, we have pleasure in acknowledging the special courtesy of the firm's representative, and in noting the spick-and-span order in which the stall is kept. Another fine show is that made by Virol, Ltd., 152 Old Street, E.C., whose well-known preparation of the red-bone marrow, malt, eggs, and fresh fruit juice is such a valuable adjunct to milk in the nutrition of infants and invalids.

Stalls for Officers to See.

Lysol has been in the front rank of coal-tar disinfectants for so long now that one is apt to forget what a big gap it filled, and what a godsend it was. Chas. Zimmerman and Co., of 9 and 10 St. Mary at Hill, E.C., show, besides lysol, a complete radium emanatorium, and all kinds of radium preparations for use in many and various disorders, as well as several other specialties in attractive forms. Horlick's Malted Milk is yet another of the really pre-eminent exhibits, and this firm deserves special commendation also for the attention which has been paid to the description of its wares in the catalogue. An excellent display of filters is that shown by Slack and Brownlow, of Manchester, and of especial merit is their hospital filter with thermometer attached, providing hot and cold sterile water of any temperature for surgical requirements. The products of Messrs. A. Wulfing and Co., 12 Chenies Street, W.C., are household words throughout the land; foramint, sanatogen, albulactin, and cystopurin are a quartette which have attained a position peculiarly secure in the affections of physicians and patients alike. The Surgical Manufacturing Co., of 85 Mortimer Street, are in the front rank of those firms which supply boxes and drums of sterilised dressings, sterilisers, operating tables, and similar conveniences for operations in private houses. They also make surgical instruments of the first quality, as those who visit their stands (131 and 132) will see for themselves. In the same transept (the east) as this exhibit are to be found specimens of the patent flush fire-resisting doors in hard woods, made by John P. White and Sons, Ltd., Pyghtle Works, Bedford. The imperative necessity of this kind of door in hospital architecture and construction has long been acknowledged; and a visit to this stand easily explains the popularity of this particular firm's goods. Yet another interesting stand in this transept, placed in the best position facing the entrance, is that of Southall Bros. and Barclay, Birmingham. The specialties of this firm are known all over the world, and are indeed a boon to humanity. Seeking new worlds to conquer, they have not only entered into the cod-liver oil business, by setting up factories at Lofoten, but have also produced a tonic food, already well known under the name of Vitafer. Galenicals of all sorts, elixirs, syrups, standardised tinctures, and many more drugs and compounds are also manufactured by this enterprising firm. Electrical apparatus of all kinds is exhibited by the Sanitas Electrical Co., Ltd., 61 New Cavendish Street, W., whose stand is one of the most interesting in the west transept. Here may be seen both the simplest and the most elaborate *x*-ray outfits, as well as appliances for diathermy and all the other newest developments of electro-therapeutics.

Some Publishing Firms.

Beyond illustrating we do not allude to Stand 105, that of The Hospital itself, further than to draw the attention of visitors to the illustrated booklet on the principal hospitals of the Metropolis which is being given away free there. Among other publishing firms we may note particularly interesting displays by Henry Frowde and Hodder and Stoughton; by H. K. Lewis; by the J. B. Lippincott Company; by Cassell and Co., Ltd.; and by the W. B. Saunders Company. There can be no doubt that medical publishing is in a particularly flourishing state just now. Not only is the quality of the works published almost uniformly high, in spite of ever-increasing quantities produced; but also the leading publishers are paying so much attention to paper and printing, illustrations, binding, and general format that the teachings of the authors are displayed to the very best advantage. Clever publishing cannot make a bad book into a good one, but it can make a good book appear better still; and of late years medical authors have had little to complain of on this score.

A brief synopsis must suffice for other exhibits. The Medical Supply Association, 167 Gray's Inn Road, W.C., for sterilisers and surgical instruments; Parke, Davis and Co. for drugs and medicaments of all sorts; Hoffmann-La Roche for omnopon, digalen, and many similar preparations; Philip Harris and Co., Ltd., for pharmaceutical products, are all worth visiting. So are the Vittel Mineral Waters stand; Humphrey Taylor and Co.'s exhibit of liqueurs and whiskies; Ingram and Royle for mineral waters; Ronuk, Ltd., for sanitary floor polish; the Holborn Surgical Instrument Co.; William Martindale for pharmaceutical products of all kinds; Claudius Ash and Co. for dental materials; Domen Belts Co., Ltd.; Hawksley and Sons, the Oxford Street instrument makers; Fairchild Bros. and Foster for their well-known fine products; the Jelloid Co.; the Maltine Manufacturing Co., Ltd.; the Antiphlogistine exhibit; Callard and Co.'s foods for obesity and diabetes; Arthur Berliner's exhibit of surgical dressings; Keen, Robinson and Co.'s barley and oatmeal foodstuffs; Duncan, Flockhart and Co.'s anæsthetics and other drugs; Lemco and Oxo at Stand No. 55; Menley and James, the makers of the glidine series of protein foods; Rouse and Co., manufacturing chemists and electro-medical instrument makers; Pearson's antiseptic substances; Salt and Sons' belts, boots, and other orthopædic appliances; Bell and Croyden's aseptic dressings and other specialties; Savory and Moore's foods, emulsions, and other digestible products; Angier's emulsion of petroleum with hypophosphites, and throat tablets; Dr. Diemel underwear; Nestlé's Swiss milk and chocolates; St. Ivel cheese and visem, made by the same firm; the Hygiama foods and beverages; Bovril; Wincarnis; Cerebos salt; the Pascon Company's soluble proteid beef extract; Dr. Jaeger's pure wool clothing and outfits; Mellin's foods and other dietetic preparations; HEWLETT'S pharmaceutical preparations; WESTON AND WESTALL'S salt and brine crystals; RADIUM, LTD., for emanation apparatus; ARMOUR AND Co., LTD., for glandular extracts and similar organo-therapeutic substances; the SCHOLL Co., LTD., for orthopædic appliances; FRANK A. ROGERS for medical sprays; the MANHU Co.'s foods for diabetic patients; KINGHAM'S Me-Malto, invalid port, and Australian burgundy: LESLIES, LTD., for surgical plasters of all kinds; W. AND J. BURROW for the natural Malvern waters; JACOBSON for oxygen apparatus; the HYGIENIC SYPHON AND CARBONATOR Co. for their well-known products; SKEFFINGTON'S invalid lifters; the ANGLO-AMERICAN PHARMACEUTICAL COMPANY'S numerous preparations; HALL'S wine, at the Stephen Smith and Co.'s stand; DOULTON'S sanitary exhibits, many of which are named after King's College Hospital; KOLYNOS dental cream; JEWSBURY AND BROWN'S aerated waters and beverages; CUXSON, GERRARD AND Co.'s surgical dressings and ligatures; the JEYES SANITARY COMPOUNDS Co., LTD., for cylein in its numerous forms; Mrs. CAMERON'S exhibit of corsets and belts; the FRIEDRICHSHALL WATER exhibit (C. Oppel and Co.); the STILLE-WERNER stand of Swedish surgical instruments; the R. B. TURNER laboratory fittings and medical glassware; BAISS BROS. AND STEVENSON'S fine chemicals and specialities; BRAND AND Co.'s meat juices and extracts; CASEIN, LTD., for milk powders and products; DAVIS' agency for catgut, horsehair, and ligatures; THOMAS CHRISTY AND Co. for their many well-known drugs and medical products; SIEMENS BROS. for electrical apparatus; ACOUSTICONS, LTD., for ear trumpets; SCHALL AND Co.'s x-ray installations; PRIDEAUX'S casein foods and soluble albumins; J. H. MOORE'S sanitary cabinets; and the DOWSING RADIANT HEAT COMPANY'S apparatus.

## Figures and Tables

**Figure f1:**